# Study on Seroprevalence and Associated Factors of Bovine Brucellosis in Selected Districts of Afar National Regional State, Afar, Ethiopia

**DOI:** 10.1155/2021/8829860

**Published:** 2021-03-03

**Authors:** Wossene Negash, Teshager Dubie

**Affiliations:** College of Veterinary Medicine, Samara University, P.O. Box 132, Samara, Russia

## Abstract

Bovine brucellosis is among the top five diseases primarily threatening both public health and livestock economy. Available data are limited to central and highland areas of the country leaving documented literature on the disease in cattle to be found hardly in pastoral and agropastoral regions of the country. As a result, the magnitude and extent of the disease remained to be investigated. A cross-sectional study design was conducted on local Afar cattle aged six months and above from February 2017 to January 2019 in selected districts of Afar region. Technically, study districts and kebeles were selected purposively whereas simple random sampling technique was applied to select cattle owners and individual animals for sample collection. An average of 8 ml whole blood was drawn of jugular vein into plain vacutainer tube using sterile needle. Using Thrusfield formula, a total of 420 blood samples were collected. The sera were tested by RBPT and CFT tests for detection of *Brucella* antibodies. Data were analyzed using Stata v14.0. Of the 420 sera tested by RBPT, 50 were positive for *Brucella* antibodies providing an overall animal level prevalence of 11.9% and those RBPT positive sera were further retested by specific and sensitive confirmatory CFT test and 24 of the retested samples had come positive for the disease providing an overall individual animal seroprevalence of 5.7% over the three districts. Of the 3 associated factors (sex, age, and district) considered, only sex had significantly associated (*P* < 0.05 : 0.036) with the disease. To estimate the strength of sex impact, odds ratio was generated using bivariate and multivariate logistic regression analyses with 95% CI and *P* < 0.05 providing OR of 2.484 (1.061–5.815) and 2.514 (1.041–6.07), respectively. Hence, the computations revealed that male cattle were 2.484 and 2.514 times more likely at higher risk for the disease as compared to their female counterparts.

## 1. Introduction

Human population depends on the domestic animals for production of meat, fat, milk, dairy products, transport, draft power, eggs production, fertilizers, and fibers [1]. In Ethiopia, more than 80% of the population is dependent on agriculture in which livestock plays a dominant role [2]. Being the major livestock component, cattle have played a crucial role throughout human the history providing draft power, milk, and meat for human consumption since domestication [3, 4].

Bovine brucellosis is considered the world's most common bacterial zoonosis [5] and highly contagious and economically important public disease. FAO, WHO, and OIE considered the disease as one of the most wide spread zoonoses in the world [6] causing economic, veterinary, and public health consequences in the developing countries [5, 7]. Within sub-Saharan Africa, many of the known infectious diseases commonly occur and are poorly controlled both in livestock and in human population. Public funds rose for the control of such infectious diseases progressively decreased over the last 20 years [8]. Brucellosis is widely spread within African countries [9] and considered by the World Health Organization as being responsible for more sickness, misery, and economic loss than any other zoonosis [10]. Bovine brucellosis is listed among the top five zoonotic diseases in Ethiopia [11].

Bovine brucellosis affects a number of species including humans, ruminants, swine, rodents, canines, and marine mammals with global distributed. Bovine brucellosis is usually caused by *Brucella abortus* and occasionally by *Brucella melitensis* and *Brucella suis* [12]. Biologically, *Brucella* species are facultative intracellular, Gram-negative, flagellated, nonmotile, oxidase positive, catalase positive, urease positive, non-spore-forming, noncapsulated, and partially acid-fast coccobacilli that lack capsules, endospores, or native plasmids. They cannot survive most disinfectants. Under appropriate conditions, Brucella can survive outside the host for extended period of time. They can remain viable in carcass and tissues for 6 months at 0°C, up to 125 days in soil and 1 year in feces [13]. Pasteurization effectively kills Brucella in milk [14].

Brucellosis is mainly transmitted through inhalation, abraded skin, or ingestion of organisms along with contaminated food and drinks. High numbers of organisms are shed in urine, milk, vaginal discharge, semen, and delivery discharges of infected animals [15, 16].

The disease is presented as an acute or persistent febrile illness with a diversity of clinical manifestations [17] having incubation period between 14 and 120 days [18]. Bovine brucellosis is clinically characterized by late term abortion, neonatal losses, infertility, reduced milk production, and death of full-term calves [19–21].

Brucellosis causes both direct and indirect losses: indirect losses include morbidity, stunting, reduced fertility, decreased milk production, lowered sale value of infected cows, lack of access to markets, restrictions of international trade of live animals and their products, disruption of local markets and direct losses include abortion, neonatal death, replacement costs, treatment costs, labor costs, emergency slaughtering of the infected animals, and stillbirths [22–25].

Bovine brucellosis has been eradicated in most developed countries through the implementation of several extensive control programs, whereas developing countries continued to experience an increasing trend of the disease because of lack of resources and coordinated control programs. Moreover, in sub-Saharan Africa, increased pastoralism and intensification of commercial livestock farms have contributed to disease impact [26].

Bovine brucellosis remains under diagnosed and under-reported in many developing countries [27] though an important bacterial disease among livestock and people in sub-Saharan Africa [28]. Epidemiology and preventive measures of brucellosis in livestock and humans are not well understood, and such information is inadequate particularly in sub-Saharan Africa [6, 8, 29].

A number of serological surveys have been documented so far indicating that brucellosis is an endemic disease in urban, periurban, highland and lowland, extensive and intensive farming, small holder farms, and ranches of the country [29–31].

Available reports reaffirmed that brucellosis is an endemic disease in Ethiopia, and researchers have established its prevalence rate in cattle in different regions of the country [32]. In the last two decades, serological studies have been reported by different scholars in Ethiopia. Accordingly, 39% by Mayer [33] in Western Ethiopia, 8.2% by Bayleyegne [34] in central part of the country, 22% by Tariku [35] in a dairy farm in Northeastern Ethiopia, 8.1% by Yilkal [36] in dairy farms in and around Addis Ababa, 11%–15% by Tekelye et al. [37], in dairy farms and ranches in southwestern Ethiopia, 7.7% by Mekonnen et al. [38] in Tigray region, 0.14% by Taddesse [39] in north Gondar zone, 0.77% by Tadele [40] in Southwestern Ethiopia, 1.11% by Yohannes et al. [41] in Addis Ababa and Sululta abattoir, 2.46% by Kassahun et al. [42] in Sidama zone of southern Ethiopia, 22% by Sintaro [43] in dairy herd of Cheffa state farm, and 5% by Berhe et al. [44] and Ibrahim et al. [45] in cattle under crop-livestock mixed farming were documented, respectively.

A study conducted by Hunduma and Regassa [46] reported the occurrence of bovine brucellosis in pastoral and agropastoral areas of East Shoa Zones of Oromia supporting the presence of the disease in pastoral and agropastoral areas of Ethiopia. Moreover, Dinka and Chala [29] reported that the prevalence of Bovine brucellosis in pastoral areas was found to be higher than agropastoral areas. However, those reports on cattle brucellosis were mainly conducted in central and northern Ethiopia and do not provide an adequate epidemiological picture of the disease in different agroecological zones and livestock production systems of the country [47], suggesting that limited studies on bovine Brucellosis have been done far in pastoral and agropastoral areas of East Africa [48, 49]. According to Dinka and Chala [29], limited data are available on cattle brucellosis in pastoral and agropastoral areas of Ethiopia despite the presence of large population of cattle. In addition to indicated scarcity of studies in pastoral areas, cattle herders in pastoral areas are in close contact with their animal, consume raw milk, and handle aborted materials further compounding brucellosis problems according to Omer et al. [50].

In general, it could be inferred that the extent and magnitude of bovine brucellosis in pastoral and agropastoral areas in general and Afar pastoral and agropastoral areas in particular have not been studied yet. Therefore, the objectives of the present study were to determine the prevalence of bovine brucellosis in selected districts of the region and to assess and identify associated factors with the disease.

## 2. Materials and Methods

The study was conducted in three selected districts of Afar region, namely, Dubti, Asaita, and Chifra. All are situated in zone one of Afar region. The Afar region is one of the nine federal states of Ethiopia located in the northeastern part of the country. The region is geographically located between 39°34' and 42°28' East Longitude and 8^o^49'and 14^o^30'North Latitude. The region comprises 5 administrative zones, 32 districts and 331 kebeles, 28 towns, and 401 rural and urban kebeles [2].

### 2.1. Study Design

A cross-sectional study design was applied to determine the seroprevalence of bovine brucellosis and associated risk factors in the selected study sites.

### 2.2. Study Population

All indigenous Afar cattle aged 6 months and above reared by pastorals and agropastorals in the selected sites were used for the study.

### 2.3. Sampling Technique and Sample Size Determination

Both randomized and purposive sampling techniques were applied for selection of study animals (cattle) and study areas. While study zone, districts, and kebeles were chosen purposively, households and study units/individual cattle were selected using simple random sampling technique. As no previous study was conducted on bovine brucellosis in cattle found in the selected areas, the present study has considered 50% expected prevalence, 95% confidence level, and 5% absolute precision or marginal error. Based on these assumptions, the total number of animals to be included in the study got determined using the Thrusfield [51] formula:

(1) $$\begin{array}{c} n=\frac{1.962\times P_{\exp}\times \left(1-P_{\exp}\right)}{d^{2}}, \end{array}$$

where *n* = required sample size, *d* = desired absolute precision, and *P*_exp_ = expected prevalence (50%).

Based on the formula, the total sample size was computed to be 384 cattle to be selected from all three districts. To minimize chance and increase precision of the outcome, the total number of study animals was increased to 420. Proportionally, a total of 128, 130, and 162 were collected from Asaita, Dubti, and Chifra districts, respectively, based on density of cattle population in the districts.

## 3. Methodology

An average of 8 ml whole blood was drawn from jugular vein of each 420 cattle into labeled plain vacutainer test tubes using *21 gauge needles*. Sera were separated from the blood into labeled cryogenic vials. Each sample container was labeled with animal ID, age, site, and sex of every animal. Labeled sera were stored at −20°C prior to serological analysis.

### 3.1. Serological Tests

Both the screening and confirmatory tests were carried out at the National Veterinary Institute, Ethiopia.

### 3.2. Screening Test or Rose Bengal Plate Test (RBPT)

Each serum was tested against Brucella agglutinin antigen using sensitive RBPT technique based on the protocol of the OIE [52, 53].

### 3.3. Confirmatory Test by Complement Fixation Test (CFT)

Those sera clearly detected positive by RBPT were further retested using the more specific confirmatory CFT according to the procedures recommended by OIE [53] using standard *Brucella abortus* antigen to detect the presence of anti-Brucella antibody in the sera.

### 3.4. Questionnaire Survey

Semistructured questionnaire were administered to selected cattle owners following verbal consent on the need of the study. For each animal sampled, questionnaire data were collected concerning age, sex, and study site to analyze the impact of these variables on the occurrence of the disease.

### 3.5. Data Management and Analysis

Relevant data were organized, coded, and entered into Microsoft Excel sheet. Organized data were transferred to Stata v14.0 [54]. Descriptive and chi-squared statistics and logistic regression analyses were employed during data analysis. Descriptive statistics will be done to determine prevalence of the disease and other frequencies. The chi-squared (*χ*^2^) statistics will be employed to determine the association between the associated factors and the disease. Both bivariate and multivariate logistic regression analyses were computed to determine the degree of strength between those associated factors and the disease (brucellosis).

## 4. Results

Descriptive statistics was employed to calculate prevalence and percentages of associated factors (sex, age, and district) with respect to the test results as summarized in (Table 1). The total number of animals sampled were 420 (*n* = 246 females and *n* = 174 males).

All (*n* = 420) sera were subjected to the screening test (Rose Bengal Plate test-RBPT) against *Brucella abortus* antigen, and 50 of them have come positive for bovine brucellosis with an overall prevalence of 11.9% with 95% CI (8.79–15.015). Those RBPT positive samples were further retested by the more specific confirmatory test of CFT of which only 24 of them were really positive for bovine brucellosis providing an overall prevalence of 5.7% (Table 2) with 95% CI (3.48–7.94).

### 4.1. Logistic Regression

To assess the impact of associated factors on the disease occurrence, chi-square (*χ*^2^) statistics was computed. Accordingly, only sex (*P* < 0.05 : 0.036) was significantly associated with the disease (bovine brucellosis). Further, using 95%CI and *P* < 0.05, unadjusted odds ratio was computed using binary logistic regression separately for each factor (sex, age, and district) to estimate the magnitude each factor could pose on the disease (Table 3). Similarly, adjusted odds ratio (AOR) was also computed simultaneously to determine the real magnitude (without compounding effect) of the factors one on the disease.

Logistic regression computations had vividly revealed that male cattle were 2.484 times more likely at risk for the disease as compared to their female counterparts.

## 5. Discussion

Brucellosis is a disease having drawn attention and concern for it causes public threat and economic losses in the cattle industry [55]. The disease can be diagnosed using several serological tests including rose Bengal test (RBPT), complement fixation test (CFT), ELISA, and others [56]. In the present study, only CFT and RBPT were applied for Brucella antibody detection and accordingly reported 5.7% and 11.9% overall individual animal level seroprevalence by CFT and RBPT, respectively. The two tests showed level of degree agreement though the RBPT test was observed to show false positivity. In epidemiological studies, the serial use of two tests is recommended to maximize the accuracy of test results [57] and the most widely used testing scheme. RBT is a highly sensitive test and could easily be applied in field conditions for the screening purpose, whereas CFT is highly specific and sensitive usually test used as a confirmatory test for detection of Brucella antibodies in the diagnosis of bovine brucellosis [55, 58].

False positive serological reactors in RBT could be due to cross-reactions with smooth lipopolysaccharide (S-LPS) antigens of other bacteria. As there has never been a history of vaccination in all of our study districts, seropositivity in this case was therefore only due to natural infection. The RBPT test result of the current study has revealed an overall prevalence of 11.9% in cattle over 3 districts in Afar region lying within the range of 10 to 15% estimated for assumed brucellosis seroprevalence for East Africa [59]. The RBPT test result had supported the evidence that, in sub-Saharan Africa, the highest incidence of brucellosis is observed in pastoral production systems [6, 8, 59].

Comparable studies have been reported by different scholars in different regions of Ethiopia. A 5% bovine brucellosis in and around Addis Ababa has been reported by Ethiopian Ministry of Agriculture (1970). According to Yilkal [36], an overall prevalence of 4.6% has been recorded in and around Addis Ababa. Similarly, Hunduma and Regassa [46] reported 4.1% in agropastoral areas of East Shoa Zone. Moreover, 4.9% by Mekonnen et al. [38] in Western Tigray and 4.63% by Mussie et al. [60] in Bahir Dar milk shed were in tandem with the present finding. In national context, studies conducted in other four African were in tandem with present record. 5.6% prevalence in Uganda by Faye et al. [61]; 6.5% in Tanzania by Kagumba and Nandokha [62]; 5.8% in Nigeria by Cadmus et al. [63]; and 5.9% in Southern Sudan by Hellman et al. [64] were recorded.

The present study has revealed higher prevalence than some previous studies. Studies lower than the present report included 1.38% by Degafu et al. [65] in Jigjiga zone of Somali region, 1.92% by Asmare et al. [66] in Sidama zone, 3.5% by Megersa et al. [47] in South Eastern Ethiopia, 1.97% by Yohannes et al. [41] in Guto-Gida district of East Wollega Zone, 2.9% by Jergefa et al. [67] in central Oromia, 0.77% by Tolosa et al. [31] in Jimma, 3.19% by Berhe et al. [44] in Tigray region, 1.2% by Haileselassie et al. [68] in Tigray region, and 0.61% by Belihu [69] in Jimma have been documented.

The higher prevalence observed in the present study could be due to related to pastoral community patterns characterized by clustering of households with their herds in camps, diversity of livestock species reared as part of coping mechanism for uncertainties and risks increase the aggregation and interaction of different animals at villages, herd size, pasture fields, and water points facilitating the transmission of the disease. The frequent migration of pastoral herds might increase the chance of coming into contact with other potentially infected herds in different neighboring areas [58, 70–73]. According to Hellman et al. [64], large herd size enhances the exposure potential, especially following abortion increasing contact in common feeding and watering points promoting transmission of Brucella organisms.

In contrast, the current CFT test result has been by far much lower than that of the previous reports of 38.7% by Rashid [74] in and around Addis Ababa, 18.4% by Bekele et al. [71] in selected farms and ranches in South Eastern Ethiopia, 24.1% by Mekonnen et al. [38] in Western Tigray, 11% by Kebede et al. [30] in smallholder farms in central Ethiopia, 33% by Corbel [75], 16.8% by Bayleyegn [34] in Arsi region, 14.2% by Taye [76] at Abernosaranch, 18.4% by Gebremariam [77] with in the dairy farms of around Addis Ababa, 19.5% by Yirgu [78] in East Shoa Ethiopia, and 16.9% by Abeje [79] in and around Bahir Dar.

On the continental scale, higher prevalence had also been reported in other African countries including 46.8% in Uganda by Kungu et al. [80]; 41% in Togo by Domingo [81]; and 14.2% in South Africa by Manhica [82].

The lower prevalence observed in this study could be attributed to the fact that the present study has covered over wide geographic coverage. Moreover, the lower prevalence in the present study would be linked to higher scorching sun pressurizing the survival of the bacterium under such high temperature [26].

The observed disparity in bovine brucellosis prevalence among different regions of Ethiopia could be attributed to various factors including differences in testing protocols, age difference, sex, pregnancy status, geographical difference, animal management practices, reproductive diseases, herd size, sample size, and the serological tests employed that would further accentuate these variations [30, 83].

Among associated factors considered under the present study, only sex was observed to have statistically significant association (*P* < 0.05) with brucellosis occurrence, whereas age and study districts had no statistically significant association with the disease (*P* > 0.05).

The study has indicated that gender has shown statistically significant association with the disease (*P* < 0.05 : 0.036). The proportion of male cattle (8.6%) was more likely to be seropositive to antibodies of Brucella species than female cattle (3.7%) (OR = 2.484; 95% CI: 1.061–5.815). Specifically, male cattle were 2.484 times more likely at risk compared to female. The current finding was in tandem with Megersa et al. [84] and Kassahun et al. [42]. The higher infection rate of male cattle in the current study could be associated to the fact that male cattle are extensively used for serving females across different herds increasing their chance of contracting the bacterium.

Apparently, an opposite outcome has been reported by different scholars indicating that female cattle were proportionally more positive for the disease compared to males. In studies conducted by Mussie et al. [60] and Asfaw et al. [85], the proportion of female cattle was more than that of males with significant association. The lower infection rate in males in these reports could be related to the fact that male cattle were kept for relatively shorter time duration in breeding herd than females and hence decreasing the chance of exposure in males [30]. Moreover, Radostits et al. [55] have shown that erythritol, a polyhydric acid found in higher concentration in the placenta and fetal fluids of females than in seminal vesicles and testis of males, can be responsible for females being more susceptible than males. More importantly, the stress associated with pregnancy and calving tends to lower immunity of female animals [86], and this might also explain the observed difference.

Unlike gender, origin or study locations were observed to hove insignificant influence on the disease. This finding was in agreement with studies conducted by Akinseye et al. [44, 87] supporting the current finding and stated that age did not play a significant role in seropositivity of brucellosis in cattle. The absence of association between the disease and study locations could be due to smaller cattle population during sample collection, good husbandry practices by pastoralists, and good public awareness about the disease.

## 6. Conclusion and Recommendations

Bovine brucellosis is a contagious, zoonotic and economically important bacterial disease of human and animals with global perspective. The disease is responsible for sickness, misery, and economic loss than any zoonotic disease. The present study has come up with a moderate record of 5.7% by CFT and 11.9% by RBPT in bovine over the three study districts. Of the three associated factors, only sex has come to be significantly associated with the disease. The current report signals a demand of changing lifestyle and husbandry practices to have more traction by government and the public.

Based on the conclusions given, the following recommendations were forwarded:

  Regional brucellosis surveillance needs to be done to control the disease
  Advanced and detailed molecular study of the bacterium needs to be done to identify the circulating strain of Brucella species in the region
  Public awareness must be made on brucellosis economic and public health impacts at a larger scale.

## Figures and Tables

**Table 1:** Descriptive variables against the disease (CFT test).

Variable	Category	CFT		*P* value
		Negative (%)	Positive (%)	
*District*	Dubti	123 (94.6)	7 (5.4)	0.644
	Asaita	118 (92.2)	10 (7.8)	
	Chifra	155 (95.7)	7 (4.3)	

*Age*	Young	119 (94.4)	7 (5.6)	0.118
	Adult	175 (97.2)	5 (2.8)	
	Old	102 (89.5)	12 (10.5)	

*Sex*	Female	237 (96.3)	9 (3.7)	0.036
	Male	159 (91.4)	15 (8.6)	

**Table 2:** Prevalence of bovine brucellosis by CFT and RBPT tests.

Test type	Samples tested	Negative samples	Positive samples	Prevalence (%)	95% CI
CFT	420	396	24	5.7	3.48–7.94
RBPT	420	370	50	11.9	8.79–15.015

**Table 3:** Summary of bivariate and multivariate logistic regression analyses.

Variable	CFT test		Binary logistic regression		Multivariate logistic regression	
	Negative	Positive	COR (95% CI)	*P* value	AOR (95% CI)	*P* value
*Age*						
Young	119	7	**1**		1	**0**
Adult	175	5	**0.486 (0.1506–1.567)**	0.227	**0.565 (0.173–1.849)**	**0.345**
Old	102	12	**2 (0.759–5.270)**	**0.161**	**2.509 (0.843–7.463)**	**0.098**

*Sex*						
Female	237	9	**1**			**0**
Male	159	15	**2.484 (1.061–5.815)**	**0.036**	**2.514 (1.041–6.07)**	**0.040**

*District*						
Dubti	123	7	**1**		**1**	**0**
Asaita	118	10	**1.489 (0.549–4.041)**	0.434	**1.387 (0.499–3.854)**	**0.530**
Chifra	155	7	**0.794 (0.271–2.323)**	0.673	**1.413 (0.427–4.668)**	**0.571**

## Data Availability

The data used to support this study are available from the corresponding authors upon request.

## Abbreviations

LMA: Livestock marketing authority
RBPT: Rose Bengal plate test
CFT: Complement fixation test
PA: Peasant association
OIE: Office International des Epizooties
PFE: Pastoralist Forum Ethiopia
CSA: Central Statistical Agency
FAO: Food and Agriculture Organization
NGOs: Nongovernmental organizations
WHO: The World Health Organization
SPSS: Statistical package for social science.
